# Outcomes of SARS-CoV-2–Positive Youths Tested in Emergency Departments: The Global PERN–COVID-19 Study

**DOI:** 10.1001/jamanetworkopen.2021.42322

**Published:** 2022-01-11

**Authors:** Anna L. Funk, Todd A. Florin, Nathan Kuppermann, Daniel J. Tancredi, Jianling Xie, Kelly Kim, Mark I. Neuman, Lilliam Ambroggio, Amy C. Plint, Santiago Mintegi, Terry P. Klassen, Marina I. Salvadori, Richard Malley, Daniel C. Payne, Norma-Jean Simon, Adriana Yock-Corrales, Jasmine R. Nebhrajani, Pradip P. Chaudhari, Kristen A. Breslin, Yaron Finkelstein, Carmen Campos, Kelly R. Bergmann, Maala Bhatt, Fahd A. Ahmad, Michael A. Gardiner, Usha R. Avva, Nipam P. Shah, Laura F. Sartori, Vikram J. Sabhaney, Kerry Caperell, Nidhya Navanandan, Meredith L. Borland, Claudia R. Morris, Iker Gangoiti, Viviana Pavlicich, Nirupama Kannikeswaran, Maren M. Lunoe, Pedro B. Rino, April J. Kam, Jonathan C. Cherry, Alexander J. Rogers, Shu-Ling Chong, Laura Palumbo, Carlos M. Angelats, Andrea K. Morrison, Maria Y. Kwok, Sarah M. Becker, Andrew C. Dixon, Naveen Poonai, Michelle Eckerle, Muhammad Wassem, Stuart R. Dalziel, Stephen B. Freedman

**Affiliations:** 1 Department of Pediatrics, Cumming School of Medicine, University of Calgary, Calgary, Alberta, Canada; 2 Department of Pediatrics, Feinberg School of Medicine, Northwestern University, Chicago, Illinois; 3 Division of Emergency Medicine, Ann and Robert H. Lurie Children’s Hospital of Chicago, Chicago, Illinois; 4 Departments of Emergency Medicine and Pediatrics, University of California, Davis School of Medicine, Sacramento; 5 Department of Pediatrics, University of California, Davis School of Medicine, Sacramento; 6 Department of Pediatrics, Harvard Medical School, Boston, Massachusetts; 7 Division of Emergency Medicine, Boston Children’s Hospital, Boston, Massachusetts; 8 Section of Emergency Medicine, Children’s Hospital Colorado, Department of Pediatrics, University of Colorado, Aurora; 9 Children’s Hospital of Eastern Ontario, Division of Emergency Medicine, Departments of Pediatrics and Emergency Medicine, University of Ottawa, Ottawa, Ontario, Canada; 10 Pediatric Emergency Department, Biocruces Bizkaia Health Research Institute, Hospital Universitario Cruces, University of the Basque Country, UPV/EHU, Bilbao, Basque Country, Spain; 11 Children’s Hospital Research Institute of Manitoba, Department of Pediatrics and Child Health, University of Manitoba, Winnipeg, Manitoba, Canada; 12 Public Health Agency of Canada, Ottawa, Ontario, Canada; 13 Division of Infectious Diseases, Boston Children’s Hospital, Harvard Medical School, Boston, Massachusetts; 14 Centers for Disease Control and Prevention, Atlanta, Georgia; 15 Data Analytics and Reporting, Division of Emergency Medicine, Ann and Robert H. Lurie Children’s Hospital of Chicago, Chicago, Illinois; 16 Hospital Nacional de Niños “Dr Carlos Sáenz Herrera”, CCSS, San José, Costa Rica; 17 St Mary’s Medical Center, West Palm Beach, Florida; 18 Division of Emergency and Transport Medicine, Children’s Hospital Los Angeles, Los Angeles, California; 19 Keck School of Medicine of the University of Southern California, Los Angeles, California; 20 Children’s National Hospital, Washington, DC; 21 Divisions of Emergency Medicine and Clinical Pharmacology and Toxicology, Department of Pediatrics Hospital for Sick Children, Toronto, Ontario, Canada; 22 Hospital Universitario Miguel Servet, Pediatric Emergency Department, Zaragoza, Spain; 23 Department of Emergency Medicine, Children’s Minnesota, Minneapolis, Minnesota; 24 Department of Pediatrics, Children’s Hospital of Eastern Ontario, Ottawa, Ontario, Canada; 25 Department of Pediatrics, Washington University School of Medicine in St Louis, St Louis, Missouri; 26 Rady Children’s Hospital, Department of Pediatrics, University of California, San Diego, San Diego, California; 27 School of Medicine Hackensack Meridian Health, Hackensack, New Jersey; 28 Division of Pediatric Emergency Medicine, Department of Pediatrics, University of Alabama at Birmingham, Birmingham; 29 Division of Pediatric Emergency Medicine, Department of Pediatrics, Children’s Hospital of Philadelphia, Philadelphia, Pennsylvania; 30 Department of Paediatrics, The University of British Columbia, Vancouver, British Columbia, Canada; 31 Norton Children’s Hospital, University of Louisville, Louisville, Kentucky; 32 Perth Children’s Hospital, Divisions of Emergency Medicine and Paediatrics, School of Medicine, University of Western Australia, Perth, Western Australia, Australia; 33 Department of Pediatrics, Division of Emergency Medicine, Emory University School of Medicine, Children’s Healthcare of Atlanta, Atlanta, Georgia; 34 Departamento de Emergencia Pediátrica, Hospital General Pediátrico Niños de Acosta Ñu, Facultad de Medicina, Universidad Privada del Pacífico, San Lorenzo, Paraguay; 35 Children’s Hospital of Michigan, Central Michigan University, Detroit; 36 UPMC Children’s Hospital of Pittsburgh, Pittsburgh, Pennsylvania; 37 Hospital de Pediatría “Prof Dr Juan P. Garrahan”, RIDEPLA, Buenos Aires, Argentina; 38 Department of Pediatrics, Division of Emergency Medicine, McMaster Children’s Hospital, Hamilton, Ontario, Canada; 39 Department of Pediatric Emergency Medicine, IWK Health Centre, Dalhousie University, Halifax, Nova Scotia, Canada; 40 Departments of Emergency Medicine and Pediatrics, University of Michigan School of Medicine, Ann Arbor; 41 Department of Emergency Medicine, KK Women’s and Children’s Hospital, Duke-NUS Medical School, SingHealth Duke-NUS Global Health Institute, Singapore; 42 ASST Spedali Civili di Brescia - Pronto soccorso pediatrico, Brescia, Italy; 43 Department of Pediatrics, Hospital Francesc de Borja, Gandia, Spain; 44 Division of Emergency Medicine, Department of Pediatrics, Medical College of Wisconsin, Milwaukee, Wisconsin; 45 Department of Emergency Medicine, New York Presbyterian Morgan Stanley Children’s Hospital, Columbia University Irving Medical Center, New York; 46 University of Utah School of Medicine and Primary Children’s Hospital, Salt Lake City, Utah; 47 University of Alberta, Stollery Children’s Hospital, Women’s and Children’s Health Research Institute, Edmonton, Alberta, Canada; 48 Child Health Research Institute, Division of Paediatric Emergency Medicine, Departments of Pediatrics, Internal Medicine, Epidemiology and Biostatistics, Schulich School of Medicine & Dentistry, London, Ontario, Canada; 49 Department of Pediatrics, University of Cincinnati College of Medicine, Cincinnati, Ohio; 50 Division of Pediatric Emergency Medicine, Cincinnati Children’s Hospital, Cincinnati, Ohio; 51 Lincoln Medical Center, Bronx, New York; 52 Children’s Emergency Department, Starship Children’s Hospital, Auckland, New Zealand; 53 Departments of Surgery and Paediatrics: Child and Youth Health, University of Auckland, Auckland, New Zealand; 54 Sections of Pediatric Emergency Medicine and Gastroenterology, Departments of Pediatrics and Emergency Medicine, Cumming School of Medicine, University of Calgary, Calgary, Alberta, Canada

## Abstract

**Importance:**

Severe outcomes among youths with SARS-CoV-2 infections are poorly characterized.

**Objective:**

To estimate the proportion of children with severe outcomes within 14 days of testing positive for SARS-CoV-2 in an emergency department (ED).

**Design, Setting, and Participants:**

This prospective cohort study with 14-day follow-up enrolled participants between March 2020 and June 2021. Participants were youths aged younger than 18 years who were tested for SARS-CoV-2 infection at one of 41 EDs across 10 countries including Argentina, Australia, Canada, Costa Rica, Italy, New Zealand, Paraguay, Singapore, Spain, and the United States. Statistical analysis was performed from September to October 2021.

**Exposures:**

Acute SARS-CoV-2 infection was determined by nucleic acid (eg, polymerase chain reaction) testing.

**Main Outcomes and Measures:**

Severe outcomes, a composite measure defined as intensive interventions during hospitalization (eg, inotropic support, positive pressure ventilation), diagnoses indicating severe organ impairment, or death.

**Results:**

Among 3222 enrolled youths who tested positive for SARS-CoV-2 infection, 3221 (>99.9%) had index visit outcome data available, 2007 (62.3%) were from the United States, 1694 (52.6%) were male, and 484 (15.0%) had a self-reported chronic illness; the median (IQR) age was 3 (0-10) years. After 14 days of follow-up, 735 children (22.8% [95% CI, 21.4%-24.3%]) were hospitalized, 107 (3.3% [95% CI, 2.7%-4.0%]) had severe outcomes, and 4 children (0.12% [95% CI, 0.03%-0.32%]) died. Characteristics associated with severe outcomes included being aged 5 to 18 years (age 5 to <10 years vs <1 year: odds ratio [OR], 1.60 [95% CI, 1.09-2.34]; age 10 to <18 years vs <1 year: OR, 2.39 [95% CI 1.38-4.14]), having a self-reported chronic illness (OR, 2.34 [95% CI, 1.59-3.44]), prior episode of pneumonia (OR, 3.15 [95% CI, 1.83-5.42]), symptoms starting 4 to 7 days prior to seeking ED care (vs starting 0-3 days before seeking care: OR, 2.22 [95% CI, 1.29-3.82]), and country (eg, Canada vs US: OR, 0.11 [95% CI, 0.05-0.23]; Costa Rica vs US: OR, 1.76 [95% CI, 1.05-2.96]; Spain vs US: OR, 0.51 [95% CI, 0.27-0.98]). Among a subgroup of 2510 participants discharged home from the ED after initial testing and who had complete follow-up, 50 (2.0%; 95% CI, 1.5%-2.6%) were eventually hospitalized and 12 (0.5%; 95% CI, 0.3%-0.8%) had severe outcomes. Compared with hospitalized SARS-CoV-2–negative youths, the risk of severe outcomes was higher among hospitalized SARS-CoV-2–positive youths (risk difference, 3.9%; 95% CI, 1.1%-6.9%).

**Conclusions and Relevance:**

In this study, approximately 3% of SARS-CoV-2–positive youths tested in EDs experienced severe outcomes within 2 weeks of their ED visit. Among children discharged home from the ED, the risk was much lower. Risk factors such as age, underlying chronic illness, and symptom duration may be useful to consider when making clinical care decisions.

## Key Points

**Question:**

What proportion of SARS-CoV-2–positive youths tested in emergency departments (ED) experience severe outcomes (ie, intensive interventions, severe organ impairment, or death) within 14 days?

**Findings:**

Among 3221 SARS-CoV-2–positive youths enrolled in a global prospective cohort study with outcome data, 3.3% had severe outcomes within 14 days. Across a subgroup of 2510 SARS-CoV-2–positive youths discharged home after testing, 0.5% had severe outcomes during the 2-week follow-up period.

**Meaning:**

The findings of this study suggest that risk factors such as age, underlying chronic illness, and symptom duration may be useful for clinicians to consider when evaluating pediatric patients with SARS-CoV-2 infection.

## Teaser

This cohort study investigates the prevalence of severe outcomes among youths aged 17 years or younger within 14 days of testing positive for SARS-CoV-2 in emergency departments in 8 countries.

## Introduction

During the early stages of the global COVID-19 pandemic, youths less than 18 years of age represented fewer than 5% of reported cases.^1,2,3,4^ These early estimates likely underreported the true number of children infected with SARS-CoV-2 because of testing capacity and the generally mild, or even asymptomatic, nature of the disease in children.^5,6^ However, the pandemic has evolved, and in the United States, youths now represent 25% of all new COVID-19 cases.^7^ Similarly, pediatric hospitalizations due to COVID-19, which increased 8-fold between May and November of 2020,^8^ have seen a further 5-fold increase between June and August 2021.^9^

Although COVID-19 is generally mild in children, severe outcomes and death do occur.^10,11,12,13^ The risk of severe outcomes among youths with SARS-CoV-2 infection is poorly understood with estimates varying considerably between study designs, settings, and regions.^1,14,15^ Studies generally include large administrative databases (ie, community based),^16^ hospitalized populations,^17^ and youths admitted to the intensive care unit (ICU).^13^ Consistently identified risk factors for severe COVID-19 in youths include young (ie, 1-3 months) or old (15-18 years) pediatric age group, male sex, and preexisting medical conditions.^18,19,20,21^ However, data from large prospective cohort studies which include youths with early or mild stages of disease seeking emergency department (ED) care are lacking.

We sought to quantify the frequency of and risk factors for severe outcomes in SARS-CoV-2–positive children enrolled in a prospective ED-based cohort study.

## Methods

### Setting, Design and Participants

The Pediatric Emergency Research Network (PERN)–COVID-19 prospective cohort study enrolled participants between March 7, 2020, and June 15, 2021, who were tested for SARS-CoV-2.^22^ Children and adolescents aged younger than 18 years who had a SARS-CoV-2 test performed because of suspected acute infection based on symptoms or exposure were eligible. Sites attempted to recruit the first 5 eligible youths each day, using a consecutive approach starting with the first test performed on chronological time. As this led to variations in the percentage of positive youths recruited across sites, the approach was revised to target the recruitment of the first 2 test-positive and 4 test-negative youths each day. For this study, we focused on youths with positive SARS-CoV-2 nucleic acid tests [eg, polymerase chain reaction (PCR)] enrolled in 38 of the PERN–COVID-19 participating EDs across 8 countries including Argentina, Canada, Costa Rica, Italy, Paraguay, Singapore, Spain, and the United States. We included those who were test-negative as a comparator group. All participants enrolled in 3 study sites in 2 countries were SARS-CoV-2 negative and were included in relevant analyses.

Enrolling sites had local institutional review board approval or established a reliance agreement with the Cincinnati Children’s Hospital Medical Center institutional review board. The legal guardians of all participants provided informed consent (written or verbal based on site) to participate in this study. This study followed the Strengthening the Reporting of Observational Studies in Epidemiology (STROBE) reporting guideline.

### Data Collection

At the time of enrollment, the participant’s guardian provided data regarding demographic characteristics, epidemiological risk factors, and clinical symptoms. Two weeks after the index ED visit, guardians were contacted via telephone, text, or email to determine if there were subsequent health care visits, treatments, or interventions received. This information was supplemented by a medical record review performed a minimum of 30 days after the index ED visit to collect data related to medical care and interventions provided, disposition, and clinical outcomes. Participants were deemed lost-to-follow-up at 14 days if 5 attempts to contact the participant’s legal guardian were unsuccessful and a medical record review was not performed. Race and ethnicity data were only available for a subset of the study population and were not reported.

### Definitions

#### Symptoms of SARS-CoV-2 Infection

Symptom duration was defined as time from reported onset of symptoms to time of ED evaluation and was grouped according to investigator consensus as 0 to 3 days, 4 to 7 days, and 8 or more days. Febrile or respiratory illness was defined as having any of the following symptoms: fever, cough, rhinorrhea, congestion, wheezing, difficulty breathing, sputum production, sore throat, or apnea.

#### SARS-CoV-2 Testing

Testing protocols varied by site and over time. Participants were classified as SARS-CoV-2 positive if they had a positive nucleic acid test (eg, PCR) during the qualifying ED encounter or in the subsequent 2 weeks in instances of repeat testing, on any of the following samples: nasal swab, nasopharyngeal swab, oropharyngeal/throat swab, and saliva. Dates of infection were grouped according to enrollment and severity trends (ie, waves).

#### Severe Outcomes

A priori, a severe outcome was defined by the occurrence of any of the following complications: cardiac or cardiovascular (cardiac arrest, cardiac ischemia, congestive heart failure, endocarditis, myocarditis, pericarditis, stroke), infectious (disseminated intravascular coagulation, mastoiditis, sepsis with bacteremia, septic shock, toxic shock syndrome), neurologic (encephalitis, meningitis), respiratory (acute respiratory distress syndrome, empyema, necrotizing or cryptogenic organizing pneumonia, pleural effusion or pneumothorax or pneumomediastinum requiring drainage, respiratory failure), and death. In the absence of documentation of one of the aforementioned events, performance of any of the following interventions was deemed to represent a severe outcome: chest drainage, extracorporeal membrane oxygenation, inotropic support, positive pressure ventilation (invasive or noninvasive), and renal replacement therapy. The diagnosis of multisystem inflammatory syndrome in children (MIS-C) and Kawasaki disease were reported as assigned by the clinical care teams and were considered severe if accompanied by one of the aforementioned diagnoses or interventions.

#### Demographics

Legal guardians of participants enrolled in the United States were provided the following race and ethnicity options from which to choose: American Indian or Alaska Native, Asian, Black or African American, Hispanic or Latino, Native Hawaiian or Other Pacific Islander, White, and other. Responses were categorized for reporting purposes as Hispanic, non-Hispanic Black, non-Hispanic other (Native American, Pacific Islander, Asian, other), and non-Hispanic White.

### Sample Size

The PERN–COVID-19 prospective cohort study originally planned to recruit up to 12 500 participants to obtain a subsample of test-positive cases that included at least 50 with severe outcomes, which would provide 93.9% power to detect when a predictive model discriminates severe from nonsevere outcomes in the larger population of test-positive youths, assuming a true C statistic of 0.70.^22^ These calculations used a variance inflation factor of 2.0 to account for model complexity as measured by degrees of freedom.^23^ As outcomes were classified in a delayed manner, we ended up having an excess number of severe outcomes prior to achieving our target sample size, which led to a termination of recruitment.

### Statistical Analysis

Pearson χ^2^ or Fisher exact tests were used to compare the prevalence of participant characteristics across age groups. All youths with ED outcome (day 0) data were included in the primary analysis. Risk differences were calculated by subtracting the cumulative incidence between categories of the following predefined variables: sex, age, and chronic conditions. Median length of hospitalization and ICU stay were compared across dichotomous strata using the Mann-Whitney test and across the 5 age groups using the Kruskal-Wallis test. Outcomes among children discharged home from the ED on day 0 and who completed 14-day follow-up were summarized using similar methods.

A multivariable logistic regression model that included the country and time period of enrollment, and accounted for clustering by site, was used to identify factors associated with severe outcomes. The following variables were specified a priori for inclusion into the model: sex, age group, chronic underlying condition as reported by the guardian (excluding asthma), history of asthma, prior pneumonia, symptoms of febrile or respiratory illness, duration of symptoms, and date of infection. From this model, we derived additional models by removing nonsignificant variables (*P* > .05) in sequence and then computing the Akaike Information Criteria (AIC) weights for each model. The model with the lowest AIC was selected as the best fit model.^24^ To evaluate associations with race and ethnicity, a similar multivariable logistic regression analysis was performed for participants recruited in EDs in the United States alone. We compared the proportion of severe outcomes by time (each month) of study enrollment using a trend test that adjusted for cluster effects.

Baseline characteristics of enrolled youths who tested SARS-CoV-2 positive and negative were compared using the χ^2^ test and R × C contingency tables as appropriate. Severe outcomes among SARS-CoV-2–negative children were compared with SARS-CoV-2–positive children overall and based on index ED visit hospitalization status stratified by country using risk difference calculation methods.

No missing data approaches were used as only 0.8% of participants lacked data required to complete primary outcome analyses. All analyses were 2-sided and conducted using STATA version 16 (StataCorp) with statistical significance defined as *P* < .05. Statistical analysis was performed from September to October 2021.

## Results

Among 10 382 study participants, 3222 (31%) tested positive for SARS-CoV-2 during the study period. Of those who tested positive, 3221 (>99.9%) had index ED visit outcomes data available for inclusion in the primary analysis, 1694 (52.6%) were male; the median (IQR) age was 3 (0-10) years. Most participants were recruited in the United States (n = 2007; 62.3%), and 484 participants (15.0%) participants reported having preexisting chronic underlying conditions (Table 1). Among SARS-CoV-2–positive symptomatic participants, respiratory symptoms were reported in 2325 (76.3%) while fever was reported in 2125 (69.7%); 172 SARS-CoV-2–positive participants (5.3%) were asymptomatic at the index visit.

**Table 1.** SARS-CoV-2 Positive Participant Demographic and Medical History Characteristics, Overall and by Symptom Status

Characteristic	Participants, No. (%)		
	All (n = 3222)	Symptomatic (n = 3048)	Asymptomatic (n = 172)
Sex			
Male	1694 (52.6)	1612 (52.9)	80 (46.5)
Female	1528 (47.4)	1436 (47.1)	92 (53.5)
Age group, y			
<1	829 (25.7)	815 (26.7)	14 (8.1)
1 to <2	425 (13.2)	411 (13.5)	14 (8.1)
2 to <5	551 (17.1)	508 (16.7)	43 (25.0)
5 to <10	576 (17.9)^a^	525 (17.2)	50 (29.1)
10 to <18	841 (26.1)^a^	789 (25.9)	51 (29.7)
Country			
Argentina	28 (0.9)	28 (0.9)	0
Canada	532 (16.5)^a^	521 (17.1)	9 (1.1)
Costa Rica	420 (13.0)	393 (12.9)	27 (15.7)
Italy	18 (0.6)	17 (0.6)	1 (0.6)
Paraguay	35 (1.1)	33 (1.1)	2 (1.2)
Singapore	30 (0.9)	17 (0.6)	13 (7.6)
Spain	152 (4.7)	148 (4.9)	4 (2.3)
United States	2007 (62.3)	1891 (62.0)	116 (67.4)
Previous pneumonia^b^	227 (7.1)	221 (7.3)	6 (3.5)
Asthma^b^	423 (13.1)	397 (13.0)	26 (15.1)
Chronic condition^b^^,^^c^	484 (15.0)	466 (15.3)	18 (10.5)
Cardiac	76 (2.4)	74 (2.4)	2 (1.2)
Developmental	87 (2.7)	84 (2.8)	3 (1.7)
Diabetes	23 (0.7)	22 (0.7)	1 (0.6)
Gastrointestinal	13 (0.4)	12 (0.4)	1 (0.6)
Hematological	67 (2.1)	66 (2.2)	1 (0.6)
Liver	5 (0.2)	5 (0.2)	0
Malignant neoplasm	24 (0.7)	23 (0.8)	1 (0.6)
Neurological	110 (3.4)	108 (3.5)	2 (1.2)
Pulmonary	52 (1.6)	49 (1.6)	3 (1.7)
Renal	47 (1.5)	43 (1.4)	4 (2.3)
Rheumatologic	8 (0.3)	7 (0.2)	1 (0.6)
Other	167 (5.2)	164 (5.4)	3 (1.7)
Clinical presentation^d^			
Respiratory symptoms^e^	2325 (72.2)	2325 (76.3)	NA
Fever	2125 (66.0)	2125 (69.7)	NA
Other systemic symptoms^f^	1860 (57.7)	1860 (61.0)	NA
Gastrointestinal^g^	1338 (43.1)	1338 (45.5)	NA
Neurological^h^	967 (30.0)	967 (31.7)	NA
Other^i^	617 (19.1)	617 (20.2)	NA
Date of infection			
Mar 7-May 31, 2020	235 (7.3)	217 (7.1)	18 (10.5)
Jun 1-Aug 31, 2020	851 (26.4)^a^	790 (25.9)	60 (34.9)
Sept 1-Nov 30, 2020	769 (23.9)	728 (23.9)	41 (23.8)
Dec 1, 2020-Feb 28, 2021	734 (22.8)	705 (23.1)	29 (16.9)
Mar 1-Jun 15, 2021	633 (19.7)^a^	608 (20.0)	24 (14.0)

Abbreviation: NA, not applicable.

^a^ Symptom status was missing for 2 male Canadian youths, one between 5 and less than 10 years of age (enrolled between March and June 2021) and one between 10 and 18 years of age (enrolled between June and August of 2020).

^b^ Missing for 4 youths (2 youths without symptom status, and 2 other youths who were asymptomatic).

^c^ Youths may have more than one specific chronic condition.

^d^ Missing for 2 Canadian youths without symptom data.

^e^ Includes cough, rhinorrhea/congestion, difficulty breathing, sore throat, chest pain, wheeze, sputum production, apnea.

^f^ Includes myalgia, arthralgia, drowsiness or lethargy, anorexia, edema of extremities, irritability.

^g^ Includes vomiting, diarrhea, abdominal pain.

^h^ Includes headache, seizure, loss of smell or taste.

^i^ Includes conjunctivitis, skin rash, oral symptoms.

**Figure 1.**
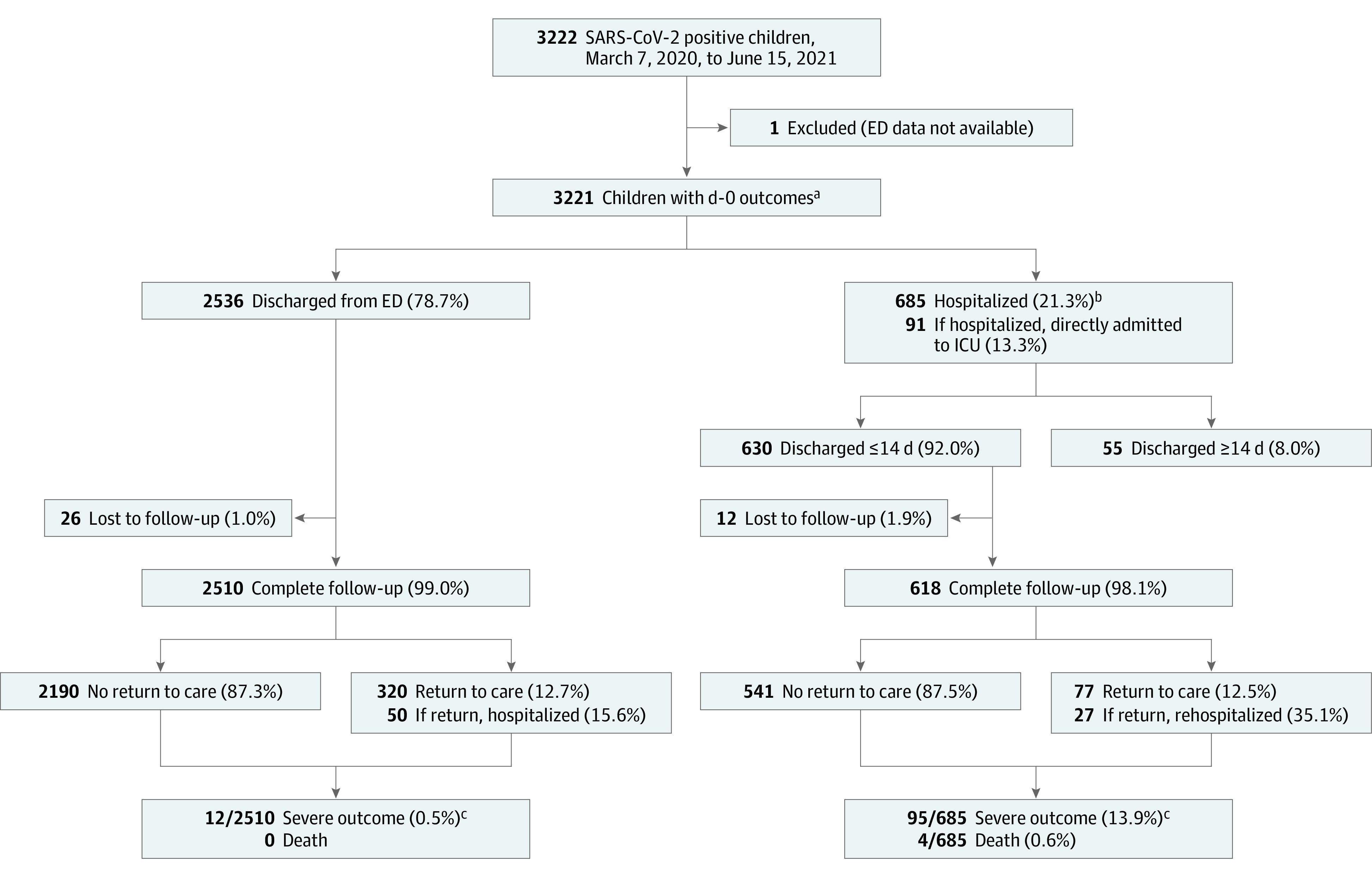
Flow of SARS-CoV-2–Positive Study Participants Including Outcomes and Follow-up ED indicates emergency department; ICU, intensive care unit. ^a^Five of 3221 youths did not test positive on day 0, but did within 14 days. ^b^Includes 8 youths transferred to other tertiary facilities. ^c^As per analysis plan, considers only youths with full 14-day follow-up.

**Figure 2.**
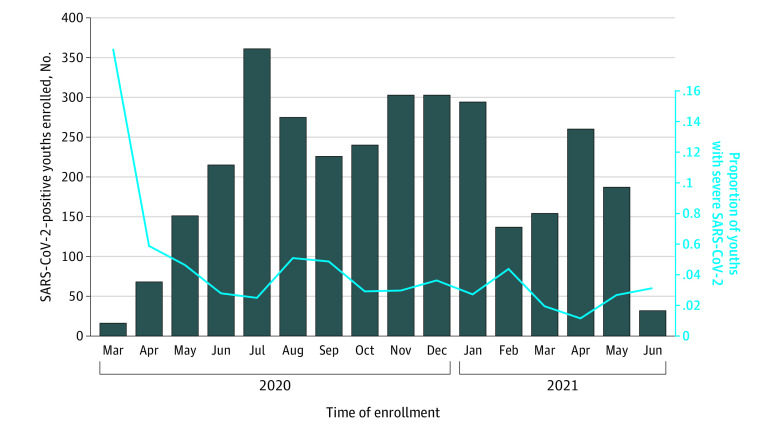
Severe Outcomes Among SARS-CoV-2–Positive Study Participants Displayed in Relation to Month of Enrollment

At the index ED visit (ie, day 0), 21.3% (n = 685) of SARS-CoV-2 positive youths were hospitalized, of whom 13.3% (91 of 685) were admitted to the ICU from the ED; (Figure 1). Within 14 days of testing positive for SARS-CoV-2 (ie, index ED visit plus 14-day follow-up period), 22.8% (95% CI, 21.4%-24.3%) of the youths (735 of 3221) had been hospitalized, 3.9% (95% CI, 3.2%-4.6%) (124 of 3221) had been admitted to ICUs, 3.3% (95% CI, 2.7%-4.0%) (107 of 3221) had severe outcomes, and 4 children (0.1%) died. Severe outcomes were most common among youths aged 10 years to less than 18 years (46 of 841; 5.5%; 95% CI, 4.0%-7.2%), and lowest among youths aged less than 1 year (14 of 828; 1.7%; 95% CI, 0.9-2.8) (Table 2). MIS-C or Kawasaki disease diagnoses were assigned to 50 of 3221 youths (1.6%; 95% CI, 1.2-2.0), 16 of whom met our severe outcome study definition. MIS-C or Kawasaki disease were most common in youths aged 5 to less than 10 years (23 of 551; 4.0%; 95% CI, 2.7-6.2); eTable 1 in Supplement 1. The proportion of youths with severe outcomes did not differ across the study enrollment period (Figure 2).

**Table 2.** Cumulative Outcomes of 3221 SARS-CoV-2–Positive Youths Within 14 Days of Index Emergency Department Visit^a^^,^^b^

Outcome	Participants, No. (%) [95% CI]									
	All children (n = 3221)	Sex		Age group, y					Chronic condition^c^	
		Male (n = 1693)	Female (n = 1528)	<1 (n = 828)	1 to <2 (n = 425)	2 to <5 (n = 551)	5 to <10 (n = 576)	10 to <18 (n = 841)	No (n = 2733)	Yes (n = 484)
Hospitalized	735 (22.8) [21.4-24.3]	406 (24.0) [22.0-26.1]	329 (21.5) [19.5-23.7]	189 (22.8) [20.0-25.8]	70 (16.5) [13.1-20.3]	120 (21.8) [18.4-25.5]	128 (22.2) [18.9-25.8]	228 (27.1) [24.1-30.3]	539 (19.7) [18.2-21.3]	195 (40.3) [35.9-44.8]
Stay, median (IQR), d^d^	3 (1-5)	3 (1-5)	3 (1-6)	2 (1-4)	2 (1-4)	3 (1-5)	3 (1-7)	3 (1-7)	2 (1-5)	3 (1-6)
ICU admission	124 (3.9) [3.2-4.6]	71 (4.2) [3.3-5.3]	53 (3.5) [2.6-4.5]	14 (1.7) [0.9-2.8]	10 (2.4) [1.1-4.3]	20 (3.6) [2.2-5.6]	20 (3.5) [2.1-5.3]	60 (7.1) [5.5-9.1]	79 (2.9) [2.3-3.6]	45 (9.3) [6.9-12.2]
Stay, median (IQR), d^e^	3 (1-5)	2 (1-4)	4 (1-7)	2 (1-4)	2.5 (0-4)	1 (0-4.5)	2 (0.5-5.5)	3 (1-6)	3 (1-5)	2 (1-7)
Severe outcome^f^	107 (3.3) [2.7-4.0]	61 (3.6) [2.8-4.6]	46 (3.0) [2.2-4.0]	14 (1.7) [0.9-2.8]	8 (1.9) [0.8-3.7]	19 (3.5) [2.1-5.3]	20 (3.5) [2.1-5.3]	46 (5.5) [4.0-7.2]	72 (2.6) [2.1-3.3]	35 (7.2) [5.1-9.9]
Severe respiratory illness or ventilatory support	73 (2.3) [1.8-2.8]	39 (2.3) [1.6-3.1]	34 (2.2) [1.5-3.1]	9 (1.1) [0.5-2.1]	5 (1.2) [0.4-2.7]	13 (2.4) [1.3-4.0]	12 (2.1) [1.1-3.6]	34 (4.0) [2.8-5.6]	43 (1.6) [1.1-2.1]	30 (6.2) [4.2-8.7]
Inotropic support	34 (1.1) [0.7-1.5]	21 (1.2) [0.8-1.9]	13 (0.9) [0.4-1.5]	2 (0.2) [0-0.9]	6 (1.4) [0.5-3.0]	2 (0.4) [0-1.3]	9 (1.6) [0.7-2.9]	15 (1.8) [1.0-2.9]	26 (1.0) [0.6-1.4]	7 (1.4) [0.6-3.0]
Severe cardiac or cardiovascular illness	9 (0.3) [0.1-0.5]	9 (0.5) [0.2-1.0]	0 [0-0.2]^g^	0 [0-0.4]^g^	1 (0.2) [0-1.3]	0 [0-0.7]^g^	2 (0.3) [0-1.2]	6 (0.7) [0.3-1.5]	7 (0.3) [0.1-0.5]	2 (0.4) [0.1-1.5]
Other severe illness, intervention, death	26 (0.8) [0.5-1.2]	13 (0.8) [0.4-1.3]	13 (0.9) [0.4-1.5]	3 (0.4) [0.1-1.1]	2 (0.5) [0.1-1.7]	7 (1.3) [0.5-2.6]	8 (1.4) [0.6-2.7]	6 (0.7) [0.3-1.5]	18 (0.7) [0.4-1.0]	8 (1.7) [0.7-3.2]

Abbreviation: ICU, intensive care unit.

^a^ No outcome information is known for the 1 youth without emergency department discharge/hospitalization information; and for 38 of 3221 youths, the^,^ outcome data were missing after day 0 (26 youths lost to follow-up who were discharged at day 0) or at the time of initial hospitalization discharge (12 lost to follow-up youths discharged from hospital within 14 days).

^b^ May extend to 4 weeks for youths hospitalized within 14 days, whose hospitalization extends past the 14-day follow-up time point.

^c^ Chronic condition missing for 4 of 3221 youths.

^d^ Length of hospitalization missing for 16 youths.

^e^ Length of ICU stay missing for 1 youth.

^f^ A youth may have multiple diagnoses and/or interventions (categories are not mutually exclusive).

^g^ 97.5% CI shown.

Among the 2510 SARS-CoV-2–positive youths discharged home from the ED index visit, with complete 14-day follow-up, 320 (12.7%; 95% CI, 11.5%-14.1%) returned to a clinic or hospital for care within 14 days due to new, worsening, or persistent symptoms (Figure 1). Specific reasons were known for 47.2% (151 of 320), the most frequent were fever (47 of 151; 31.1%) and cough (42 of151; 27.8%) (eTable 2 in Supplement 1). Fifty of these 2510 (2.0%; 95% CI, 1.5%-2.6%) children were eventually hospitalized. Twelve children (0.5%; 95% CI, 0.3%-0.8%) had severe outcomes and none died (1-sided 97.5% CI, 0%-0.2%) (Figure 1 and eTable 3 in Supplement 1). Among children discharged home, severe outcomes were more common among those with self-reported chronic underlying conditions (risk difference, 1.7%; 95% CI, 0.6%-4.0%).

Multivariable logistic regression identified the following risk factors for severe outcomes: aged 5 to 18 years (age 5 to <10 years vs <1 year: odds ratio [OR], 1.60 [95% CI, 1.09-2.34]; age 10 to <18 years vs <1 year: OR, 2.39 [95% CI 1.38-4.14]), having a preexisting chronic illness (OR, 2.34 [95% CI, 1.59-3.44]), previous episode of pneumonia (OR, 3.15 [95% CI, 1.83-5.42]), and presenting to the hospital 4 to 7 days after symptom onset (vs starting 0-3 days before seeking care: OR, 2.22 [95% CI, 1.29-3.82]) (Table 3). Country also was associated with severe outcomes, with the United States as the referent group, the risk was lower in Canada and Spain and increased in Costa Rica. Among youths from the United States, similar risk factors were identified; and race and ethnicity were not associated with severe outcomes (eTable 4 in Supplement 1).

**Table 3.** Association of Demographic Factors and Medical History With Severe Outcomes Among the 3141 SARS-CoV-2–Positive Youths With Complete Covariate Data^a^^,^^b^

	Participants, No./Total No.	aOR (95% CI)	*P* value
Country^c^			
Canada	2/529	0.11 (0.05-0.23)	<.001
Costa Rica	19/420	1.76 (1.05-2.96)	.03
Paraguay	2/35	1.43 (0.78-2.61)	.25
Spain	3/152	0.51 (0.27-0.98)	.05
United States	81/2005	[Reference]	[Reference]
Sex			
Female	46/1448	[Reference]	[Reference]
Male	61/1586	1.32 (0.83-2.12)	.24
Age category, y			
<1	14/806	[Reference]	[Reference]
1 to <2	8/416	1.00 (0.47-2.13)	>.99
2 to <5	19/537	1.66 (0.95-2.90)	.07
5 to <10	20/553	1.60 (1.09-2.34)	.02
10 to <18	46/829	2.39 (1.38-4.14)	.002
Chronic condition			
No	72/2664	[Reference]	[Reference]
Yes	35/477	2.34 (1.59-3.44)	<.001
Previous pneumonia			
No	84/2921	[Reference]	[Reference]
Yes	23/220	3.15 (1.83-5.42)	<.001
Asthma			
No	89/2727	[Reference]	[Reference]
Yes	18/414	0.65 (0.39-1.08)	.10
Symptom duration before testing, d			
Asymptomatic	9/156	2.31 (0.81-6.59)	.12
0-3	31/1369	[Reference]	[Reference]
4-7	35/702	2.22 (1.29-3.82)	.004
≥8	11/16	2.13 (0.86-5.28)	.10
Unknown	21/698	1.44 (0.84-2.45)	.18
Date of index ED visit			
Mar-May 2020	14/204	1.87 (0.63-5.51)	.26
Jun-Aug 2020	29/790	[Reference]	[Reference]
Sep-Nov 2020	27/733	0.90 (0.53-1.53)	.69
Dec 2020-Feb 2021	25/701	1.02 (0.43-2.42)	.97
Mar 2021-Jun 2021	12/606	0.75 (0.37-1.48)	.40

Abbreviations: aOR, adjusted odds ratio; ED, emergency department.

^a^ A total of 3145 youths with any outcome data were included from the 5 countries with any severe outcomes, and data were missing for 1 or more variables for 4 further youths (2 from Canada, 2 from the United States).

^b^ This table reflects the final parsimonious model.

^c^ Severe outcomes did not occur outside of these countries.

Outcome data were available for 7156 of 7160 SARS-CoV-2–negative youths enrolled in the cohort (eFigure in Supplement 1). SARS-CoV-2–negative and –positive youths differed by country, enrollment time period, age, presence of chronic underlying conditions, previous pneumonia episode, and index ED visit symptomatology (eTable 5 in Supplement 1). The proportion of SARS-CoV-2–negative youths who experienced severe outcomes within 14 days did not differ from that among the 3221 SARS-CoV-2–positive youths (risk difference, 0.6%; 95% CI, −0.1%-1.4%); eTable 6 in Supplement 1. The risk of a severe outcome was higher among hospitalized SARS-CoV-2 positive children (risk difference, 3.9%; 95% CI, 1.1%-6.9%) overall and within the United States specifically (risk difference, 6.0%; 95% CI, 2.1%-10.3%).

## Discussion

In this global cohort study, after 14 days of prospective follow-up for 3221 youths who tested positive for SARS-CoV-2 infection, 23% had been hospitalized, 3% experienced severe outcomes, and 4 children died. We identified the following risk factors for severe outcomes: aged greater than 5 years, having a preexisting chronic illness, previous episode of pneumonia, and presenting to the hospital 4 to 7 days after symptom onset. Among 2510 SARS-CoV-2–positive youths discharged home, only 0.5% had severe outcomes during the follow-up period. Although the overall proportion of SARS-CoV-2–negative youths who experienced severe outcomes did not differ from that among test-positive youths, among hospitalized children, those who were SARS-CoV-2 positive were more likely to experience severe outcomes.

Retrospective multicenter and database studies of ambulatory and hospital-based pediatric cohorts have provided varying estimates of the risk of severe outcomes among youths infected by SARS-CoV-2. Although a recent international database study that included 242 158 children and adolescents with COVID-19 reported that only 1.3% of infected youths were hospitalized and that 30-day deaths were below reportable limits (ie, <5 per database),^16^ a US hospital ED and inpatient database analysis reported that among 43 465 youths with COVID-19, 10% were hospitalized and 3% had severe illness.^25^ Similarly, while 20% of youths hospitalized in a US-based retrospective cohort study had severe or very severe disease,^17^ in a prospective Canadian study of 150 hospitalized SARS-CoV-2–positive youths, 50% had severe or critical illness.^26^ Thus, an understanding of the study population is crucial to interpreting risk estimates. Our study population provides a risk estimate for youths brought for ED care. Our lower estimate of severe disease likely reflects our stringent definition which required the occurrence of complications or specific invasive interventions.

As with other pediatric COVID-19 studies, we identified that older age, and having a preexisting chronic condition, were risk factors for severe outcomes.^18,19,21,25,26,27^ In contrast with some other studies, we did not find that very young infants were at a higher risk for severe outcomes.^18,21,27^ In some studies where very young infants were identified as being at higher risk, the outcome of interest was hospitalization or ICU admission,^21,27^ whereas we required specific intensive care interventions or complications. As indications for ICU admission differ substantially by time and place, it is a strength of our study that we did not consider ICU admission in isolation to be an indicator of severe disease.

Although asthma has been suggested as a risk factor for severe illness in youths with COVID-19,^18^ our study, as well as a registry-based study in the United States, did not confirm this association.^27^ To the best of our knowledge, no other studies have identified symptom duration prior to hospital presentation as a risk factor for severe pediatric COVID-19. As effective therapeutics for youths with detected SARS-CoV-2 are infrequently administered, it is unlikely that this association indicates a beneficial effect of earlier presentation to care. Rather, this may reflect the natural history of infection in youths, with symptom progression appearing between 4 and 7 days being more likely to lead to both hospital presentation and severe outcomes. Previous studies have shown that compared with White non-Hispanic persons in the United States, Black race and Hispanic ethnicity are associated with increased test-positivity and hospitalizations,^28,29,30,31^ including among children.^27^ However, our and other analyses that adjust for age, comorbidities, and socioeconomic indicators, among both youths and adults, have not confirmed an increased risk of intensive care, severity, and mortality due to COVID-19 among these groups.^29,32,33,34,35^

Our findings from the prospective follow-up of the subgroup of SARS-CoV-2–positive youths who were discharged home from their index ED visits reflect the natural history of mild-to-moderate pediatric SARS-CoV-2 infection. Among these 2510 youths, approximately 13% returned to either a clinic or hospital for care within 14 days due to worsening, persistent or new symptoms, 2% were hospitalized, 0.5% had severe outcomes, and none died. These findings align with a recent retrospective cohort study that included 45 US-based children’s hospitals and 15 913 ED COVID-19 encounters which led to discharge among which 10% were associated with a repeat ED encounter within 30 days.^17^

### Strengths and Limitations

This study had some strengths and limitations. A strength of our study was our ability to compare outcomes among SARS-CoV-2 test-positive children to similar, albeit not identical, test-negative children recruited prospectively, prior to the occurrence of outcomes. Although overall the risk of severe outcomes did not differ between those who were test-positive relative to those who were test-negative, the risk of severe outcomes was higher among hospitalized SARS-CoV-2 positive children when compared with those who were test-negative. This finding does not stand in isolation; in a large retrospective study that included 242 158 children with COVID-19 and 2 084 180 with influenza infection, hospitalization (5- to 13-fold), hypoxemia, and pneumonia were more frequent in those with COVID-19.^16^

As recruitment of study participants took place in EDs, our findings overestimate the risk of severe outcomes among SARS-CoV-2-positive youths and should not be interpreted to reflect the risk faced by community-based cases; rather, they are meant to provide an estimate of this risk among an ED-screened pediatric population. Sites were asked to enroll participants consecutively based on time of testing, however, the proportion of potentially eligible participants consenting to participate likely varied by site and period, which challenges the representativeness of our study population to reflect all youths tested for SARS-CoV-2 in pediatric EDs. Similarly, although participating EDs were given the same study protocol for recruitment, various factors including regional case definitions, screening criteria, and testing capacity, were not controlled by the study and could have differed by site and period. Thus, 5% of our SARS-CoV-2–positive participants were asymptomatic—most of whom were tested as they were positive contacts of known cases or as part of routine screening procedures. To account for this concern, our multivariable logistic regression model adjusted for country and period. Because participating EDs were located in academic pediatric institutions, we cannot generalize our results to all community EDs nor can we generalize to countries beyond those included in our analysis. Finally, as testing for variants of concern was not universal, we were unable to include circulating variants in our model.

## Conclusions

The findings from this large global prospective cohort study support a growing body of literature on the risk of severe outcomes and factors associated with these events in SARS-CoV-2–infected youths. Among youths who sought care in the ED, these events occurred in approximately 3% of SARS-CoV-2–positive youths, with the risk varying by age, history of underlying conditions, symptom duration and country. Our findings suggest a low risk of severe outcomes among youths discharged to home. However, among hospitalized SARS-CoV-2–positive youths, the risk of severe outcomes exceeds that of test-negative youths.

## Notes

Supplement 1. **eTable 1.** Cumulative Outcomes, Up to 14 Days Following the Index ED Visit, Among 3221 SARS-CoV-2 Positive Children **eTable 2.** Reasons for Return to Care Among 320 SARS-CoV-2 Positive Children Discharged on day 0 With Complete Follow-up **eTable 3.** Cumulative Outcomes, Up to 14 Days Following the Index ED Visit, Among 2510 SARS-CoV-2 Positive Children Who Were Discharged to Home From the Index Emergency Department Visit **eTable 4.** Association of Demographic Factors (Including Race and Ethnicity) and Medical History With Severe Outcomes Among the 2001 SARS-CoV-2 Positive Children From the United States With Complete Covariate Data **eTable 5.** Comparison of Baseline Characteristics, SARS-CoV-2 Positive and SARS-CoV-2 Negative Participants Enrolled in the PERN-COVID-19 Cohort Study **eTable 6.** Severe Outcomes in SARS-CoV-2 Negative and SARS-CoV-2 Positive Children, Stratified by Chronic Condition and Country of Enrollment **eFigure.** Flow of SARS-CoV-2 Negative Study Participants Including Outcomes and Follow-up

Supplement 2. **Nonauthor Collaborators.** Pediatric Emergency Research Network–COVID-19 Study Team
