# Supplementary material for: Outcomes of SARS-CoV-2–Positive Youths Tested in Emergency Departments: The Global PERN–COVID-19 Study
Source: JAMA Netw Open. 2022 Jan 11;5(1):e2142322. doi: 10.1001/jamanetworkopen.2021.42322 (PMC8753506; doi:10.1001/jamanetworkopen.2021.42322)
Supplement: Supplement 1. — eTable 1. Cumulative Outcomes, Up to 14 Days Following the Index ED Visit, Among 3221 SARS-CoV-2 Positive Children eTable 2. Reasons for Return to Care Among 320 SARS-CoV-2 Positive Children Discharged on day 0 With Complete Follow-up eTable 3. Cumulative Outcomes, Up to 14 Days Following the Index ED Visit, Among 2510 SARS-CoV-2 Positive Children Who Were Discharged to Home From the Index Emergency Department Visit eTable 4. Association of Demographic Factors (Including Race and Ethnicity) and Medical History With Severe Outcomes Among the 2001 SARS-CoV-2 Positive Children From the United States With Complete Covariate Data eTable 5. Comparison of Baseline Characteristics, SARS-CoV-2 Positive and SARS-CoV-2 Negative Participants Enrolled in the PERN-COVID-19 Cohort Study eTable 6. Severe Outcomes in SARS-CoV-2 Negative and SARS-CoV-2 Positive Children, Stratified by Chronic Condition and Country of Enrollment eFigure. Flow of SARS-CoV-2 Negative Study Participants Including Outcomes and Follow-up [file jamanetwopen-e2142322-s001.pdf]

Supplemental Online Content

Funk AL, Florin TA, Kuppermann N, et al; Pediatric Emergency Research Network-COVID-19 Study Team. Outcomes of SARS-CoV-2–positive youths tested in emergency departments: the global PERN–COVID-19 study. *JAMA Netw Open.* 2022;5(1):e2142322. doi:10.1001/jamanetworkopen.2021.42322

eTable 1. Cumulative Outcomes, Up to 14 Days Following the Index ED Visit, Among 3221 SARS-CoV-2 Positive Children

eTable 2. Reasons for Return to Care Among 320 SARS-CoV-2 Positive Children Discharged on day 0 With Complete Follow-up

eTable 3. Cumulative Outcomes, Up to 14 Days Following the Index ED Visit, Among 2510 SARS-CoV-2 Positive Children Who Were Discharged to Home From the Index Emergency Department Visit

eTable 4. Association of Demographic Factors (Including Race and Ethnicity) and Medical History With Severe Outcomes Among the 2001 SARS-CoV-2 Positive Children From the United States With Complete Covariate Data

eTable 5. Comparison of Baseline Characteristics, SARS-CoV-2 Positive and SARS-CoV-2 Negative Participants Enrolled in the PERN-COVID-19 Cohort Study

eTable 6. Severe Outcomes in SARS-CoV-2 Negative and SARS-CoV-2 Positive Children, Stratified by Chronic Condition and Country of Enrollment

eFigure. Flow of SARS-CoV-2 Negative Study Participants Including Outcomes and Follow-up

This supplemental material has been provided by the authors to give readers additional information about their work.

eTable 1: Cumulative outcomes, up to 14 days[a] following the index ED visit, among 3221[b] SARS-CoV-2 positive children. Data are n *(%) [95%CI or one-sided 97.5%CI]* unless otherwise stated.

	All children	Sex		Age group					Chronic condition[c]	
		Male	Female	< 1 yr	1-<2 yr	2-<5 yr	5-<10 yr	10-<18 yr	No	Yes
	N=3221	N=1693	N=1528	N=828	N=425	N=576	N=551	N=842	N=2733	N=484
Severe respiratory illness or ventilatory support	73 (2.3) [1.8-2.8]	39 (2.3) [1.6-3.1]	34 (2.2) [1.5-3.1]	9 (1.1) [0.5-2.1]	5 (1.2) [0.4-2.7]	13 (2.4) [1.3-4.0]	12 (2.1) [1.1-3.6]	34 (4.0) [2.8-5.6]	43 (1.6) [1.1-2.1]	30 (6.2) [4.2-8.7]
Acute respiratory distress syndrome	20 (0.6)	12 (0.7)	8 (0.5)	5 (0.6)	1 (0.2)	5 (0.9	2 (0.3)	7 (0.8)	13 (0.5)	7 (1.4)
Respiratory failure	40 (1.2)	23 (1.4)	17 (1.1)	5 (0.6)	4 (0.9)	6 (1.1)	7 (1.2)	18 (2.1)	21 (0.8)	19 (3.9)
Pleural effusion or pneumothorax[e]	6 (0.2)	4 (0.2)	2 (0.1)	0 (0)	0 (0)	0 (0)	2 (0.3)	4 (0.5)	4 (0.1)	2 (0.4)
Positive pressure ventilation	43 (1.3)	22 (1.3)	21 (1.4)	3 (0.4)	3 (0.7)	8 (1.5)	9 (1.6)	20 (2.4)	23 (0.8)	20 (4.1)
Chest drainage procedure	6 (0.2)	4 (0.2)	2 (0.1)	0 (0)	0 (0)	2 (0.4)	1 (0.2)	3 (0.4)	3 (0.1)	3 (0.6)
Inotropic support	34 (1.1) [0.7-1.5]	21 (1.2) [0.8-1.9]	13 (0.9) [0.5-1.5]	2 (0.2) [0-0.9]	6 (1.4) [0.5-3.0]	2 (0.4) [0-1.3]	9 (1.6) [0.7-2.9]	15 (1.8) [1.0-2.9]	26 (1.0) [0.6-1.4]	8 (1.7) [0.7-3.2]
Cardiovascular illness	9 (0.3) [0.1-0.5]	9 (0.5) [0.2-1.0]	0 (0) [0-0.2]	0 (0) [0-0.4]	1 (0.2) [0-1.3]	0 (0) [0-0.7]	2 (0.3) [0-1.2]	6 (0.7) [0.3-1.5]	7 (0.3) [0.1-0.5]	2 (0.4) [0.1-1.5]
Cardiac arrest	1 (0.03)	1 (0.06)	0 (0)	0 (0)	1 (0.2)	0 (0)	0 (0)	0 (0)	1 (0.04)	0 (0)
Heart failure	2 (0.06)	2 (0.1)	0 (0)	0 (0)	0 (0)	0 (0)	0 (0)	2 (0.2)	1 (0.04)	1 (0.2)
Stroke	1 (0.03)	1 (0.06)	0 (0)	0 (0)	0 (0)	0 (0)	0 (0)	1 (0.1)	1 (0.04)	0 (0)
Myocarditis, endocarditis, pericarditis	6 (0.2)	6 (0.4)	0 (0)	0 (0)	0 (0)	0 (0)	2 (0.3)	4 (0.5)	5 (0.2)	1 (0.2)
Other severe illness, intervention	22 (0.7) [0.4-1.0]	11 (0.6) [0.3 -1.2]	11 (0.7) [0.4-1.3]	3 (0.4) [0.1-1.1]	2 (0.5) [0.1-1.7]	6 (1.1) [0.4-2.4]	5 (0.9) [0.3-2.0]	6 (0.7) [0.3-1.5]	18 (0.7) [0.4-1.0]	4 (0.8) [0.2-2.1]
Meningitis or encephalitis	5 (0.2)	3 (0.2)	2 (0.1)	1 (0.1)	0 (0)	2 (0.4)	1 (0.2)	1 (0.1)	5 (0.2)	0 (0)

© 2022 Funk AL et al. *JAMA Network Open.*

Septic shock	11 (0.3)	4 (0.2)	7 (0.5)	1 (0.1)	2 (0.5)	2 (0.3)	3 (0.5)	3 (0.4)	10 (0.4)	1 (0.2)
Sepsis with bacteremia	2 (0.1)	1 (0.06)	1 (0.07)	0 (0)	1 (0.2)	1 (0.2)	0 (0)	0 (0)	2 (0.1)	0 (0)
Renal replacement therapy	5 (0.2)	4 (0.2)	1 (0.07)	1 (0.1)	0 (0)	0 (0)	2 (0.3)	2 (0.2)	2 (0.1)	3 (0.6)
Death	4 (0.1) [0.04-0.3]	2 (0.1) [0.02-0.5]	2 (0.1) [0.02-0.5]	0 (0) [0-0.6]	0 (0) [0-1.1]	1 (0.2) [0-1.1]	3 (0.5) [0.1-1.7]	0 (0) [0-0.6]	0 (0) [0-0.2]	4 (0.8) [0.3-2.3]
**MIS-C or Kawasaki**	50 (1.6) [1.2-2.0]	30 (1.8) [1.2-2.5]	20 (1.3) [0.8-2.0]	2 (0.2) [0-0.9]	4 (0.9) [0.3-2.4]	12 (2.2) [1.1-3.8]	23 (4.0) [2.7-6.2]	9 (1.1) [0.5-2.0]	45 (1.6) [1.2-2.2]	5 (1.0) [0.3-2.4]
Severe outcome	16 (0.5)	11 (0.6)	5 (0.3)	0	2 (0.5)	2 (0.3)	7 (1.3)	5 (0.6)	15 (0.5)	1 (0.2)

[a] May extend to 4 weeks for children hospitalized within 2 weeks, whose hospitalization extends past the 2-week follow-up time-point.
[b] No outcome information is known for the 1/3222 children without any emergency department discharge/hospitalization information
[c] Chronic condition is missing for 5/3221
[d] A child may have multiple diagnoses / interventions (categories are not mutually exclusive)
[e] requiring drainage

© 2022 Funk AL et al. *JAMA Network Open.*

eTable 2: Reasons for return to care among 320 SARS-CoV-2 positive children discharged on day 0 with complete follow-up.

Reason for return	n (%)
**Return to ED/hospital, reason unknown[a]**	**151/320 (47.2)**
**Worsening or persistent symptoms general**	**18/320 (5.6)**
**Specific reason given for return[b]**	**151/320 (47.2)**
Fever	47/151 (31.1)
Cough	42/151 (27.8)
Other reason (presumed linked to COVID-19)[c]	28/151 (18.5)
Difficulty breathing	25/151 (16.6)
Rash (not specific to hands/feet)	21/151 (13.9)
Vomiting	19/151 (12.6)
Diarrhea	10/151 (6.6)
Abdominal pain	10/151 (6.6)
Dehydration	6/151 (4.0)
Rash on hands	6/151 (3.5)
Rash on feet	2/151 (1.2)

[a]Question asked as 'return related to initial reason for ED visit or possible complications of illness?', 36/151 of these children were hospitalized.
[b]Children may have more than one reason noted.
[c]Symptom presumed by guardian/caregiver to be possibly linked to COVID-19.

© 2022 Funk AL et al. *JAMA Network Open.*

eTable 3: Cumulative outcomes, up to 14 days[a] following the index ED visit, among 2510[b] SARS-CoV-2 positive children who were discharged to home from the index emergency department visit. Data are n *(%) [95%CI or one-sided 97.5% CI]* unless otherwise stated.

	All children	Sex		Age group					Chronic condition[c]	
		**Male**	**Female**	**< 1 yr**	**1-<2 yr**	**2-<5 yr**	**5-<10 yr**	**10-<18 yr**	**No**	**Yes**
	**N=2510**	**N=1306**	**N=1204**	**N=647**	**N=362**	**N=432**	**N=449**	**N=620**	**N=2204**	**N=303**
**Returned to care** (new symptoms, worsening condition, or persistent symptoms)	320 (12.7) [11.5-14.1]	167 (12.8) [11.0-14.7]	153 (12.7) [10.9-14.7]	107 (16.5) [13.8-19.6]	49 (13.5) [10.2-17.5]	44 (10.2) [7.5-13.4]	37 (8.2) [5.9-11.2]	83 (13.4) [10.8-16.3	270 (12.3) [10.9-13.7]	50 (16.5) [12.5-21.2]
**Hospitalized**	50 (2.0) [1.5-2.6]	28 (2.1) [1.4- 3.1]	22 (1.8) [1.1-2.8]	16 (2.5) [1.4-4.0]	9 (2.5) [1.1-4.7]	8 (1.9) [0.8-3.6]	4 (0.9) [0.2-2.3]	13 (2.1) [1.1-3.6]	36 (1.6) [1.1-2.3]	14 (4.6) [2.5- 7.6]
Median stay in days [IQR][d]	3.0 (1.0, 5.2)	3.0 (1.0, 4.0)	3.0 (2.0, 9.0)	1.0 (0, 2.5)	3.5 (0.3, 5.5)	3.0 (1.0, 6.0	5.0 (1.0, 11.0)	5.0 (3.0, 9.8)	3.0 (1.0, 4.0)	7.0 (2.0, 10.3)
**ICU admission**	8 (0.3) [0.2, 0.7]	1 (0.1) [0-0.5]	7 (0.6) [0.3-1.3]	0 [0-0.7]	0 [0-1.2]	1 (0.2) [0.01-1.5]	1 (0.2) [0.01-1.4]	6 (0.2) [0.01-1.4]	4 (0.2) [0.06-0.5]	4 (1.3) [0.4, 3.6]
Median stay in days [IQR]	6.0 (3.0, 11.0	3.0 (NA)	7.0 (3.5, 11.0)	NA	NA	2.0 (NA)	11.0 (NA)	6.0 (3.5, 9.5)	3.4 (2.3, 7.0)	11.0 (6.0, 11.0)
**Severe outcome[e]**	12 (0.5) [0.2-0.8]	3 (0.2) [0-0.7]	9 (0.7) [0.3-1.4]	2 (0.3) [0-1.1]	0 [0-1.0]	2 (0.5) [0.1-1.7]	1 (0.2) [0-1.2]	7 (1.1) [0.5-2.3]	6 (0.3) [0.1-0.6]	6 (2.0) [0.7-4.3]
Severe respiratory illness[f]	9 (0.4)	2 (0.2)	7 (0.6)	2 (0.3)	0	1 (0.2)	1 (0.2)	5 (0.8)	4 (0.2)	5 (1.7)
Inotropic support	1 (0.04)	0	1 (0.1)	0	0	1 (0.2)	0		1 (0.05)	0
Myocarditis	1 (0.04)	1 (0.1)	0							
Sepsis with bacteremia	1 (0.04)	0	1 (0.1)	0	0	1 (0.2)	0	0	1 (0.05)	0
Coagulation abnormality	1 (0.04)	0	1 (0.1)	0	0	0	0	1 (0.2)	0	1 (0.3)

[a] May extend to 4 weeks for children hospitalized within 2 weeks, whose hospitalization extends past the 2 week follow-up time-point.
[b] Outcomes were censored (and not presented here) for 26/2536 children who were discharged at Day 0 but were lost-to-follow-up.
[c] Chronic condition information is missing for 3/2536.
[d] Missing length of stay for 8 hospitalized children.
[e] A child may have multiple diagnoses / interventions (categories are not mutually exclusive).
[f] Includes use of positive pressure ventilation or diagnosis of respiratory failure or diagnosis of acute respiratory distress syndrome .

© 2022 Funk AL et al. *JAMA Network Open.*

eTable 4: Association of demographic factors (including race and ethnicity) and medical history with severe outcomes among the 2001 SARS-CoV-2 positive children from the United States with complete covariate data. The following table reflects the final parsimonious model.

	n/N	Adjusted Odds Ratio	95% Confidence Interval	P-value
**Sex**				
Female	32/975	**ref**		
Male	49/1026	**1.65**	0.96 – 2.83	0.07
**Age category**				
< 1 year	10/478	**ref**		
1 – <2 years	7/223	**1.52**	0.69 – 3.35	0.30
2 - <5 years	11/302	**1.51**	0.72 – 3.19	0.28
5 - <10 years	13/353	**1.56**	0.99 – 2.45	0.06
10 - <18 years	40/645	**2.69**	1.32 – 5.47	**0.006**
**Ethnicity and race[a]**				
Hispanic	30/789	**ref**		
Non-Hispanic Black	30/736	**1.00**	0.47 – 2.13	>0.99
Non-Hispanic White	14/324	**0.92**	0.50 – 1.69	0.79
Non-Hispanic Other	7/152	**1.26**	0.38 – 4.20	0.71
**Chronic condition[b]**				
No	53/1696	**ref**		
Yes	28/305	**2.51**	1.56 – 4.06	**<0.001**
**Previous pneumonia[b]**				
No	62/1838	**ref**		
Yes	19/163	**2.86**	1.57 – 5.19	**0.001**
**Asthma[b]**				
No	66/1670	**ref**		
Yes	15/331	**0.60**	0.31 – 1.15	0.13
**Symptom duration before testing**				
Asymptomatic		**1.35**	0.48 – 3.79	0.57
0-3 days	25/886	**ref**		
4-7 days	30/484	**2.41**	1.28 – 4.55	**0.007**
8+ days	8/144	**1.79**	0.70 – 4.60	0.23
Unknown	18/487	**1.21**	0.66 – 2.21	0.54
**Date of SARS-CoV-2 infection**				
Mar 7 – May 31, 2020	14/195	**1.72**	0.58 - 5.06	0.33
Jun 1 – Aug 31, 2020	22/538	**ref**		
Sept 1 – Nov 30, 2020	20/474	**0.80**	0.42 – 1.52	0.49
Dec 1, 2020 – Feb 28, 2021	15/491	**0.57**	0.29 – 1.16	0.12
Mar 1 – June 15, 2021	10/303	**0.68**	0.30 – 1.54	0.36

[a] Race and ethnicity data were missing for 4 children from the United States.
[b] Chronic condition, previous pneumonia, and asthma data were missing for 2 children from the Unites States.

© 2022 Funk AL et al. *JAMA Network Open.*

eTable 5: Comparison of baseline characteristics, SARS-CoV-2 positive and SARS-CoV-2 negative participants enrolled in the PERN-COVID-19 cohort study.

	SARS-CoV-2 positive N=3222	SARS-CoV-2 negative N=7160	P-value
**Male**	1694 (52.6)	3833 (53.5)	0.37
**Age group**			<0.001
<1 year	829 (25.7)	1426 (20.1)	
1 - <2 year	425 (13.2)	1291 (18.0)	
2 - <5 years	551 (17.1)	1616 (22.6)	
5 - <10 years	576 (17.9)	1371 (19.1)	
10 - <18 years	841 (26.1)	1446 (20.2)	
**Country**			<0.001
Argentina	28 (0.9)	61 (0.9)	
Australia	0 (0)	199 (2.8)	
Canada	532 (16.5)	2402 (33.5)	
Costa Rica	420 (13.0)	516 (7.2)	
Italy	18 (0.6)	3 (0)	
New Zealand	0 (0)	106 (1.5)	
Paraguay	35 (1.1)	21 (0.3)	
Singapore	30 (0.9)	40 (0.6)	
Spain	152 (4.7)	259 (3.6)	
United States	2007 (62.3)	3553 (49.6)	
**Previous pneumonia**[a]	227 (7.1)	767 (10.7)	<0.001
**Asthma**[b]	423 (13.1)	1011 (14.1)	0.17
**Chronic condition**[c,d]	484 (15.0)	1512 (21.2)	<0.001
**Clinical presentation**[e]			
Asymptomatic	172 (5.3)	377 (5.3)	0.89
Symptomatic	3048 (94.7)	6768 (94.7)	
Fever	2125 (66.0)	4289 (59.9)	<0.001
Respiratory[f]	2325 (72.2)	4143 (57.9)	<0.001
Gastrointestinal[g]	1338 (43.1)	3383 (47.2)	<0.001
Other[h]	2176 (67.5)	5343 (74.6)	<0.001
**Date of infection**			<0.001
Mar 7 – May 31, 2020	235 (7.3)	2079 (29.0)	
Jun 1 – Aug 31, 2020	851 (26.4)	2749 (38.4)	
Sept 1 – Nov 30, 2020	769 (23.9)	923 (12.9)	
Dec 1, 2020 – Feb 28, 2021	734 (22.8)	719 (10.0)	
Mar 1 – June 15, 2021	633 (19.7)	690 (9.6)	

[a] Missing for 4 SARS-CoV-2 positive children, and 15 SARS-CoV-2 negative children.
[b] Missing for 4 SARS-CoV-2 positive children, and 15 SARS-CoV-2 negative children.
[c] Missing for 4 SARS-CoV-2 positive children, and 16 SARS-CoV-2 negative children.
[d]Children may have more than one specific chronic condition.
[e]Missing for 2 SARS-CoV-2 positive children, and 15 SARS-CoV-2 negative children.
[f]Includes cough, rhinorrhea/congestion, difficulty breathing, sore throat, chest pain, wheeze, sputum production, apnea.
[g]Includes vomiting, diarrhea, abdominal pain.

© 2022 Funk AL et al. *JAMA Network Open.*

[h] Includes headache, seizure, loss of smell or taste, myalgia, arthralgia, drowsiness or lethargy, anorexia, oedema of extremities, irritability, conjunctivitis, skin rash, oral symptoms.

© 2022 Funk AL et al. *JAMA Network Open.*

eTable 6: Severe outcomes in SARS-CoV-2 negative and SARS-CoV-2 positive children, stratified by hospitalization status on day 0.

	All children			Hospitalized on Day 0			Discharged on Day 0[a]		
	SARS-CoV-2 status			SARS-CoV-2 status			SARS-CoV-2 status		
	Positive	Negative	P-Value	Positive	Negative	P-Value	Positive	Negative	P-Value
**All children**	107/3221 (3.3)	195/7156 (2.7)	0.09	95/685 (13.9)	182/1820 (10.0)	0.006	12/2510 (0.5)	13/5336 (0.2)	0.09
**Country** Argentina	0/28 (0)	2/61 (3.3)	>0.99	0/25 (0)	2/54 (3.7)	>0.99	0/3	0/7	-
Australia	-	0/199	-	-	0/70	-	-	0/129	-
Canada	2/532 (0.4)	20/2402 (0.8)	0.41	1/33 (3.0)	15/171 (8.8)	0.48	1/498 (0.2)	5/2227 (0.2)	>0.99
Costa Rica	19/420 (4.5)	13/516 (2.5)	0.09	19/127 (15.0)	13/118 (11.0)	0.36	0/293	0/398	-
Italy	0/18 (0)	0/3 (0)	-	0/12	0/1	-	0/6	0/2	-
New Zealand	-	0/106	-	-	0/14	-	-	0/92	-
Paraguay	2/35 (5.7)	3/21 (14.3)	0.35	2/12 (16.7)	3/13 (23.1)	>0.99	0/23	0/8	-
Singapore	0/30 (0)	0/40 (0)	-	0/30	0/5	-	-	0/35	-
Spain	3/152 (2.0)	6/259 (2.3)	>0.99	3/45 (6.7)	6/131 (4.6)	0.70	0/107	0/128	-
United States	81/2007 (4.0)	151/3553 (4.2)	0.70	70/401 (17.5)	143/1243 (11.5)	0.002	11/1606 (0.7)	8/2310 (0.4)	0.16

[a]Excludes 26 and 31 SARS-CoV-2 positive and negative children, respectively, who were lost to follow-up).
[b]Chronic condition information missing for 4 SARS-CoV-2 positive children and 12 SARS-CoV-2 negative children.

© 2022 Funk AL et al. *JAMA Network Open.*

eFigure. Flow of SARS-CoV-2 negative study participants including outcomes and follow-up.

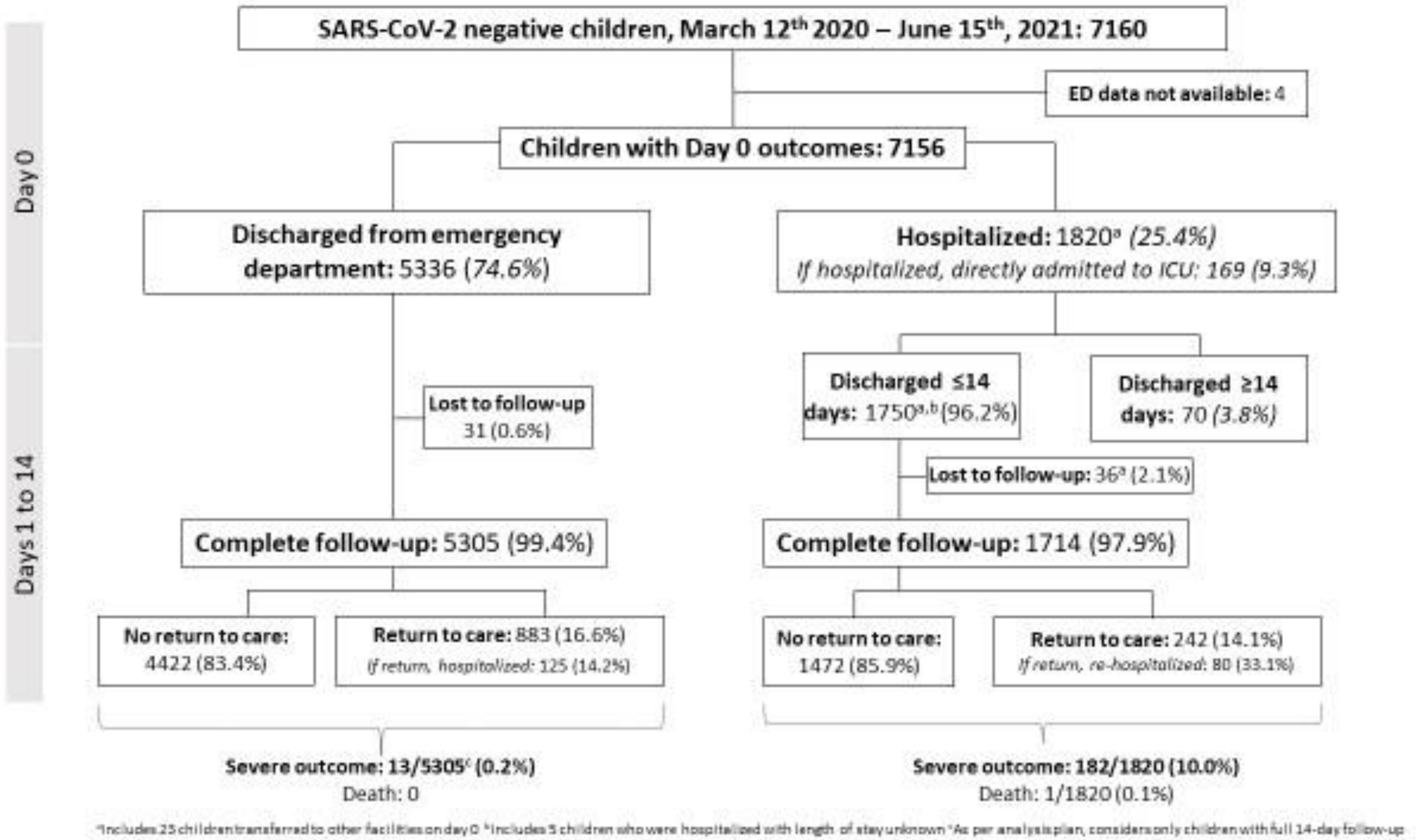

© 2022 Funk AL et al. *JAMA Network Open.*
