# Supplementary material for: Outcomes of SARS-CoV-2–Positive Youths Tested in Emergency Departments: The Global PERN–COVID-19 Study
Source: JAMA Netw Open. 2022 Jan 11;5(1):e2142322. doi: 10.1001/jamanetworkopen.2021.42322 (PMC8753506; doi:10.1001/jamanetworkopen.2021.42322)
Supplement: Supplement 2. — Nonauthor Collaborators. Pediatric Emergency Research Network–COVID-19 Study Team [file jamanetwopen-e2142322-s002.pdf]

Supplemental Online Content: Nonauthor Collaborators

*Indicates required information. Only first name, last name, and suffix will appear in PubMed.

*Group Name(s): Pediatric Emergency Research Network-COVID-19 Study Team							
***First Name and Middle Initial(s)**	***Last Name**	***Suffix (eg, Jr, III)**	Academic Degrees	Institution	Location (city, state/province, country)	Role or Contribution, eg, chair, principal investigator	Group (if more than 1 Group listed in the byline) and/or Subgroup (eg, Steering Committee)
Jessica	Gómez-Vargas			Hospital Nacional de Niños Dr. Carlos Saenz Herrera	San José, Costa Rica	Site Investigator	
Bethany	Lerman			Hospital for Sick Children	Toronto, ON Canada	Research Coordinator	
James	Chamberlain			Children's National Medical Center	Washington, DC, United States	Site Investigator	
Adebola	Owolabi			Children's National Medical Center	Washington, DC, United States	Research Assistant	
Camilla	Schanche-Perret Gentil			Children's National Medical Center	Washington, DC, United States	Research Coordinator	
Sofie	Ringold			Children's Hospital Los Angeles	Los Angeles, CA, United States	Research Coordinator	
Jocy	Perez			Children's Hospital Los Angeles	Los Angeles, CA, United States	Research Assistant	
Heidi	Vander Velden			Children's Minnesota	Minneapolis, MN, United States	Research Coordinator	
Tyrus	Crawford			Children's Hospital of Eastern Ontario	Ottawa, ON, Canada	Research Coordinator	
Steven E	Schultz			Palm Beach Children's Hospital	West Palm Beach, FL, United States	Site Investigator	
Kimberly	Ross			Palm Beach Children's Hospital	West Palm Beach, FL, United States	Research Coordinator	
Kathy	Monroe			Children's of Alabama	Birmingham, AL, United States	Site Investigator	
Karly	Stillwell			British Columbia Children's Hospital	Vancouver, BC, Canada	Research Coordinator	
Jillian	Benedetti			Lurie Children's Hospital	Chicago, IL, United States	Research Coordinator	
Sharon	O'Brien			Perth Children's Hospital	Perth, Australia	Research Coordinator	

Supplemental Online Content: Nonauthor Collaborators

*Indicates required information. Only first name, last name, and suffix will appear in PubMed.

*First Name and Middle Initial(s)	*Last Name	*Suffix (eg, Jr, III)	Academic Degrees	Institution	Location (city, state/province, country)	Role or Contribution, eg, chair, principal investigator	Group (if more than 1 Group listed in the byline) and/or Subgroup (eg, Steering Committee)
Kyle	Pimenta			UC Davis Medical Center	Sacramento, CA, United States	Research Coordinator	
Amia	Andrade			UC Davis Medical Center	Sacramento, CA, United States	Research Coordinator	
Adam	Isacoff			Norton Children's Hospital	Louisville, KY, United States	Site Investigator	
Kendra	Sikes			Norton Children's Hospital	Louisville, KY, United States	Research Coordinator	
Nina	Gold			Sanzari Children's Hospital	Hackensack, NJ, United States	Site Investigator	
Kathleen	Reichard			Sanzari Children's Hospital	Hackensack, NJ, United States	Site Investigator	
Maureen	Nemetski			Sanzari Children's Hospital	Hackensack, NJ, United States	Site Investigator	
Pavani	Avva			Sanzari Children's Hospital	Hackensack, NJ, United States	Site Investigator	
Rakesh	Mistry			Children's Hospital of Colorado	Aurora, CO, United States	Site Investigator	
Shanon	Young			UPMC Children's Hospital of Pittsburgh	Pittsburgh, PA, United States	Research Coordinator	
Marlena	Cook			CHOP Children's Hospital of Philadelphia	Philadelphia, PA, United States	Research Coordinator	
Virginia	Gómez Barrena			Hospital Infantil Universitario Miguel Servet	Zaragoza, Spain	Site Investigator	
Sandra	Castejón Ramírez			Hospital Infantil Universitario Miguel Servet	Zaragoza, Spain	Site Investigator	
María T	García Castellanos			Hospital Infantil Universitario Miguel Servet	Zaragoza, Spain	Site Investigator	
Emma	Patterson			Boston Children's Hospital	Boston, MA, United States	Research Coordinator	
Anicka	Samuel			Boston Children's Hospital	Boston, MA, United States	Research Coordinator	
Redjana	Carciurmaru			McMaster Children's Hospital	Hamilton, ON, Canada	Research Coordinator	
Eleanor	Fitzpatrick			IWK Health Center	Halifax, NS, Canada	Research Coordinator	

Supplemental Online Content: Nonauthor Collaborators

*Indicates required information. Only first name, last name, and suffix will appear in PubMed.

*First Name and Middle Initial(s)	*Last Name	*Suffix (eg, Jr, III)	Academic Degrees	Institution	Location (city, state/province, country)	Role or Contribution, eg, chair, principal investigator	Group (if more than 1 Group listed in the byline) and/or Subgroup (eg, Steering Committee)
Megan	Bonisch			Starship Children's Hospital	Auckland, New Zealand	Research Coordinator	
Bruce	Wright			Stollery Children's Hospital	Edmonton, AB, Canada	Site Investigator	
Mithra	Sivakumar			Stollery Children's Hospital	Edmonton, AB, Canada	Research Coordinator	
Patricia	Candelaria			Stollery Children's Hospital	Edmonton, AB, Canada	Research Coordinator	
Vincent	Cervantes			University of Michigan	Ann Arbor, MI, United States	Research Coordinator	
Shaminy	Manoranjithan			Children's Healthcare of Atlanta-EMORY	Atlanta, GA, United States	Research Coordinator	
Nabeel	Khan			Children's Healthcare of Atlanta-EMORY	Atlanta, GA, United States	Research Coordinator	
Toni	Harbour			Primary Children's Hospital-Intermountain Healthcare	Salt Lake City, UT,	Research Coordinator	
Usha	Sethuraman			Wayne State University (Childrens Hospital of Michigan)	Detroit, MI,	Site Investigator	
Priya	Spencer			Wayne State University (Childrens Hospital of Michigan)	Detroit, MI,	Research Coordinator	
Neha	Gupta			Wayne State University (Childrens Hospital of Michigan)	Detroit, MI,	Research Coordinator	
Amira	Kamboj			Wayne State University (Childrens Hospital of Michigan)	Detroit, MI,	Research Assistant	
Gael	Muanamputu			Wayne State University (Childrens Hospital of Michigan)	Detroit, MI,	Research Assistant	
Guillermo	Kohn Loncarica			Hospital de Pediatría Prof. Dr. Juan P. Garrahan	Buenos Aires, Argentina	Site Investigator	
Eugenia	Hernández			Hospital de Pediatría Prof. Dr. Juan P. Garrahan	Buenos Aires, Argentina	Site Investigator	
Ana	Dragovetzky			Hospital de Pediatría Prof. Dr. Juan P. Garrahan	Buenos Aires, Argentina	Site Investigator	
Isabel	Beneyto Ferré			Hospital Universitario Francesc de Borja	Gandia, Spain	Site Investigator	
Sylvia	Torres			Children's Hospital of Wisconsin	Milwaukee, WI, United States	Research Coordinator	
Joseph	Zorc			CHOP Children's Hospital of Philadelphia	Philadelphia, PA, United States	Site Investigator	
Rebecca	Haber			CHOP Children's Hospital of Philadelphia	Philadelphia, PA, United States	Research Coordinator	
Ren Mee	Hiong			KK Women's and Children's Hospital	Singapore	Research Coordinator	

Supplemental Online Content: Nonauthor Collaborators

*Indicates required information. Only first name, last name, and suffix will appear in PubMed.

***First Name and Middle Initial(s)**	***Last Name**	***Suffix (eg, Jr, III)**	Academic Degrees	Institution	Location (city, state/province, country)	Role or Contribution, eg, chair, principal investigator	Group (if more than 1 Group listed in the byline) and/or Subgroup (eg, Steering Committee)
Dianna	Sri Dewi			KK Women's and Children's Hospital	Singapore	Research Coordinator	
Gary	Joubert			London Health Sciences Centre	London, ON, Canada	Site Investigator	
Kamary	Coriolano Dasilva			London Health Sciences Centre	London, ON,	Research Coordinator	
Julie	Ochs			Columbia - Morgan Stanley Childrens Hospital of NY-Presbyterian	New York City, NY, United States	Research Coordinator	
Alberto	Arrighini			PRONTO SOCCORSO PEDIATRICO - A.S.S.T. Spedali Civili di Brescia	Brescia, Italy	Site Investigator	
Camilla	Dallavilla			PRONTO SOCCORSO PEDIATRICO - A.S.S.T. Spedali Civili di Brescia	Brescia, Italy	Site Investigator	
Andrea	Kachelmeyer			Cincinnati Children's Hospital Medical Center	Cincinnati, OH, Canada	Research Coordinator	
Daisy	Marty Placencia			Lincoln Medical Center	New York City, NY, United States	Site Investigator	
